# Analytical and Clinical Evaluation of a Chemiluminescent Immunoassay to Detect Serum Chitinase-3-like Protein 1 in HBV-Related Liver Diseases

**DOI:** 10.1155/2024/6688819

**Published:** 2024-01-24

**Authors:** Yanqiang Liao, Se Peng, Lesheng Huang, Zhong Li, Jian Hu, Rui Xu, Wenzhi Tang, Jialing Zhuang

**Affiliations:** ^1^Department of Laboratory Medicine, Doumen Qiaoli Hospital of Traditional Chinese Medicine, Zhuhai 519125, China; ^2^Department of Laboratory Medicine, Guangdong Provincial Hospital of Chinese Medicine, Zhuhai Hospital, Zhuhai 519015, China; ^3^Department of Radiology, Guangdong Provincial Hospital of Chinese Medicine, Zhuhai Hospital, Zhuhai 519015, China; ^4^Zhuhai Seesheen Medical Technology Company Limited, Zhuhai 519060, China

## Abstract

Serum chitinase-3-like protein 1 (CHI3L1) is a diagnostic marker for liver diseases, such as hepatocellular carcinoma (HCC). Herein, we aimed to evaluate the analytical performance of a chemiluminescent immunoassay (CLIA) for the quantitative detection of CHI3L1 and its application in hepatitis B virus (HBV)-related liver diseases. The CLIA for CHI3L1 detection presented good analytical performance, with a linear range of 1.50–2000.00 ng/mL and a detection limit of 0.98 ng/mL. To evaluate its clinical application, serum CHI3L1 levels were detected in 82 patients with chronic hepatitis B (CHB) and in 21 healthy controls. The patients with CHB and HCC had higher CHI3L1 levels than the healthy controls and the patients with CHB without HCC. However, CHI3L1 levels did not change significantly with the increase in liver fibrosis stages. The area under the receiver operating characteristic curve for the diagnosis of HBV-related HCC was 0.808, representing a moderate diagnostic value. Correlation analysis revealed a significant association between CHI3L1 and alpha-fetoprotein (AFP) levels, the fibrosis-4 (FIB-4) index, and the aspartate aminotransferase-to-platelet ratio index (APRI). In conclusion, compared with currently reported methods for CHI3L1 detection, the CLIA has a high sensitivity, a wide linear range, and an acceptable accuracy, precision, and reference intervals, making it valuable in the diagnosis of HBV-related HCC.

## 1. Introduction

Hepatitis B virus (HBV) is the most common cause of liver diseases in China, which can lead to acute and chronic hepatitis B (CHB), hepatic fibrosis, liver cirrhosis (LC), and hepatocellular carcinoma (HCC) [1]. In the clinical setting, timely, accurate, and comprehensive diagnoses are critical to reducing the progression of HBV-related liver diseases. As the gold standard for the diagnosis of liver diseases, liver biopsy has some limitations in clinical application because of its invasive nature [2]. Clinical symptoms, physical examination, imaging screening, and routine laboratory indicators can be insufficiently specific to detect HCC [2]. Therefore, a noninvasive, safe, and specific method would have crucial clinical application in HBV-related liver diseases.

Chitinase-3-like protein 1 (CHI3L1, YKL-40 protein) is a glycoprotein that plays a role in numerous diseases, such as arthritis, idiopathic pulmonary fibrosis, and liver diseases [3–5]. In particular, CHI3L1 is abundantly expressed in liver tissue and is mainly involved in inflammation and tissue remodeling [6]. In clinical applications, studies have shown that CHI3L1 levels were significantly elevated in liver diseases and increased with the severity of the disease [5, 7], which was also emphasized in the “Guidelines on the Management of Hepatic Encephalopathy in Cirrhosis” [8]. Furthermore, serum CHI3L1 has been recommended as a noninvasive marker for liver diseases. A meta-analysis showed that serum CHI3L1 served as an excellent marker to diagnose liver fibrosis, in which the pooled diagnostic values were significantly higher than the clinical indicators such as FibroScan, the aspartate aminotransferase-to-platelet ratio index (APRI), and the fibrosis-4 (FIB-4) index [9]. Moreover, the “Guidelines on the Prevention and Treatment in Chronic Hepatitis B” emphasized the predictive role of CHI3L1 in HBV-related liver diseases [2]. These studies indicated that CHI3L1 could be used as a biomarker for the diagnosis, staging, and prognosis of HBV-related liver diseases.

Various analytical methods have been used to detect serum CHI3L1 [10–12]. The enzyme-linked immunosorbent assay (ELISA) is the most frequently used method to quantify serum CHI3L1 levels; however, its time-consuming operations have limited its clinical application [10]. Later, fluorescent immunoassay methods with simple operation processes were developed. However, the results of the magnetic bead fluorescent immunoassay (MB-FIA) and the fluorescence immunochromatography assay (FICA) were not accurate enough [11, 12]. With the characteristics of a short determination time and high specificity, the chemiluminescent immunoassay (CLIA) has been increasingly used in routine clinical applications [13]. Considering the important clinical value of a CLIA for the rapid and accurate quantification of CHI3L1, it is necessary to explore its analytical performance and clinical application. In this study, we evaluated the analytical performance of a CLIA to quantitatively detect CHI3L1 and the value of CHI3L1 in diagnosing HBV-related liver diseases.

## 2. Materials and Methods

### 2.1. Reagents and Instruments

An automatic CLIA analyzer (iFlash3000-A, Shenzhen Yahui Long Biological Technology Co., Ltd., Shenzhen, China) was used to detect serum CHI3L1 levels. The CHI3L1 assay kits (CLIA method) (lot no. 20210201), supporting reagents, including three calibrators (0, 80.05, and 1070.48 ng/ml) and two calibrators at different concentrations from another lot (lot no. 20210601; 70.90 and 864.90 ng/ml), and other materials for the instrument, were also purchased from Shenzhen Yahui Long Biological Technology Co., Ltd. The CHI3L1 assay was a sandwich immunoassay using a direct chemiluminometric technique. According to the manufacturer's instructions, we loaded the samples, reagents, and other materials related to the CHI3L1 assay into the iFlash3000-A. At least 5 *μ*l of the sample was measured for each determination. Then, the automatic CLIA analyzer performed operations “incubation-washing-signal triggering” and measurement. The CLIA assay procedure from sample addition to result acquisition was performed in less than 20 min.

### 2.2. Performance Verification

#### 2.2.1. Linear Range

Following the Clinical and Laboratory Standards Institute's (CLSI) EP6-A guidelines, three levels of traceable calibration solution (lot no. 20210201; 0, 80.05, and 1070.48 ng/mL) were analyzed in duplicate to obtain the calibration curve for CHI3L1 [14]. Then, to verify the linear range of 1.50–2000.00 ng/mL, as suggested by the manufacturer's instructions, low- and high-value serum specimens were prepared. The low- and high-value specimens had concentrations that covered the linear range as far as possible. A series of sample concentrations were tested by mixing low (L)- and high(H)-value plasma in certain proportions (5L, 4L + 1H, 3L + 2H, 2L + 3H, 1L + 4H, and 5H). Two replicates of each sample were tested. A scatter diagram and regression analysis were then performed. A linear correlation coefficient of more than 0.99 met the industry-recognized standards.

#### 2.2.2. Functional Sensitivity: Limit of Detection

Following the CLSI's EP17-A guidelines, the limit of detection (LOD) was determined from an assay of 10 replicates of the zero-level calibration solution (lot no. 20210201; 0 ng/mL) [15]. Based on the calibration curve, the chemiluminescence value of the mean + 2 standard deviations (SD) at the zero-concentration point was converted into the corresponding concentration, which was the LOD concentration.

#### 2.2.3. Accuracy

This procedure followed the EP10-A3 guidelines published by the CLSI [16]. In this study, the detection of two concentrations of traceable calibration solutions (lot no. 20210601; 70.90 and 864.90 ng/mL) was repeated twice a day and for a total of 5 days. The accuracy was accepted when the bias was not more than 12.5%.

#### 2.2.4. Precision

Following the CLSI's EP15-A2 guidelines, two levels of mixed fresh serum were used [17]. The intraanalysis precision was assessed by calculating the coefficient of variation (CV) (%) of each level three times per run in one day. These samples were analyzed three times for five days to obtain the interanalysis comparison data. The precision was acceptable when the CV was not greater than 10.0%.

#### 2.2.5. Reference Intervals

Following the CLSI's EP28-A3c guidelines [18], 21 specimens collected from healthy individuals were analyzed for their CHI3L1 levels. On the condition that less than two specimens exceeded the reference interval suggested by the manufacturer, the validation was accepted.

### 2.3. Clinical Samples

Patients with hepatitis B were diagnosed according to the “2019 Guidelines for the Prevention and Treatment of Chronic Hepatitis B.” The exclusion criteria included the following: (1) aged <18 years; (2) lack of liver biopsy results; (3) lack of sufficient samples for the detection of CHI3L1; (4) complicated with severe diseases; and (5) pregnant or lactating women. The selection criteria for healthy controls were mainly based on negative results for hepatitis B surface antigen (HBsAg) and normal liver function. The sample size required more than 100 cases to meet the requirements of CLSI's EP9-A3 [19]. Peripheral blood samples were centrifuged at 3000 × *g* for 10 minutes to obtain the supernatant (serum). Serum samples were stored at −80°C for subsequent use. The routine clinical indicators were provided from clinical electronic databases. Alanine aminotransferase (ALT), aspartate aminotransferase (AST), alkaline phosphatase (ALP), gamma-glutamyl transferase (GGT), total protein (TP), total bilirubin (TBIL), lactate dehydrogenase (LDH), and alpha-fetoprotein (AFP) levels were measured by using an automatic biochemistry analyzer (Roche Cobas c 602/702, Roche Diagnostics GmbH, Mannheim, Germany). The platelet (PLT) parameters were detected using a Sysmex XN-9000 automatic hematology analyzer (Sysmex, Kobe, Japan). The APRI and FIB-4 indices were obtained by using the following formulae [20]:

(1) $$\begin{array}{c} \text{FIB}-4=\frac{\text{age}\left(\text{years}\right)\times \text{AST}\left(\text{IU}/L\right)}{\left(\text{PLT}\left(10^{9}/L\right)\right.\times \left(\sqrt{\text{ALT}\left(\text{IU}/L\right)}\right.}\times 100,\\ \text{ARPI}=\frac{\left(\text{AST}\left(\text{IU}/L\right)\right)/\left(\text{ULN}*\text{of AST}\left(\text{IU}/L\right)\right.}{\text{PLT}\left(10^{9}/L\right)}\times 100, \end{array}$$

where ULN is the upper limit of normal.

This study was approved by the Ethics Committee of the Guangdong Provincial Hospital of Chinese Medicine (ZE2021-084-01). Written informed consent was obtained from the patients and healthy volunteers.

### 2.4. Statistical Analysis

All statistical analyses were performed using SPSS 23 (IBM Corp., Armonk, NY, USA). Normally distributed data are shown as the mean ± SD; otherwise, M (P25 ∼ P75) was used. Comparisons of multiple groups were carried out by using a one-way analysis of variance (ANOVA) followed by a Bonferroni test for normally distributed and equal variance data; otherwise, a Kruskal–Wallis test was used. The receiver operating characteristic (ROC) curve was plotted to assess the diagnostic accuracy. We used Pearson's correlation analysis to assess whether the CHI3L1 concentration correlated with the clinical indicators. *P* values of <0.05 were considered statistically significant.

## 3. Results

### 3.1. Performance Verification

#### 3.1.1. Analysis of the Linear Range

**Figure 1:**
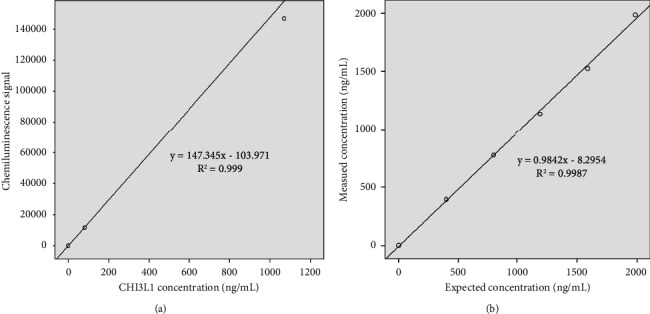
Analysis of the CHI3L1 calibration curve and the linear range. (a) Calibration curve for the CLIA assay to detect CHI3L1. (b) Scatter diagram of the measured concentration and the expected concentration. Each point represents the mean value of replicate measurements (*n* = 2). CHI3L1: chitinase-3-like protein 1.

The CHI3L1 standard curve equation was *y* = 147.345x − 103.971, *R*^2^=0.999 (Figure 1(a) and raw data in Supplementary Table 1). The expected concentration and the experimental results are summarized in Table 1. As shown in Figure 1(b), the linear correlation coefficient was 0.9987, which was greater than 0.9900. These results indicated that the linear range of 1.50 ng/mL–2000.00 ng/mL stated in the reagent instructions was acceptable (Table 1 and Figure 1(b)).

#### 3.1.2. Analysis of the LOD

The LOD concentration was determined as 0.98 ng/mL (raw data in Supplementary Table 2). Therefore, the detection range was between 0.98 and 2000.00 ng/mL. In addition, several immunoassay methods for serum CHI3L1 levels are summarized in Table 2. Compared with the methods detailed in previous reports [10–12], CLIA presented a shorter detection time, a wider linear range, and a lower detection limit.

#### 3.1.3. Analysis of Accuracy

The biases of the low-concentration calibration solution and high-concentration calibration solution between the instrumental concentration and the theoretical concentration were 0.48% and 0.08%, respectively, which were both less than 12.5% (Table 3 and raw data in Supplementary Table 3).

#### 3.1.4. Analysis of the Precision of the Assay

As shown in Table 4, the intraassay CVs for levels 1 and 2 were 1.81% and 2.76%, respectively; the interassay CVs were 7.76% and 1.61%, respectively; and the total precision CVs were 7.91% and 2.88%, respectively, which were all less than the acceptable range of not more than 10.0%, indicating that the precision of the testing kit was acceptable (raw data in Supplementary Table 4).

#### 3.1.5. Validation of Biological Reference Intervals

In samples from 21 healthy individuals, the results were all <79.0 ng/mL (raw data in Supplementary Table 5). Thus, the reference interval passed the verification standard.

### 3.2. Serum CHI3L1 Detection in Clinical Samples

**Figure 2:**
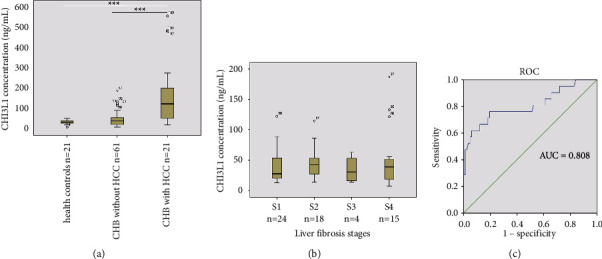
Serum CHI3L1 detection in clinical samples. (a) Comparison of CHI3L1 levels among the three groups (^*∗∗∗*^*P* < 0.001). (b) Comparison of CHI3L1 levels among the different stages of liver fibrosis. (c) The ROC curve for the ability of CHI3L1 to diagnose in HBV-related HCC. The number by the dot in the graphs is the sample number. CHI3L1: chitinase-3-like protein 1; CHB: chronic hepatitis B; HCC: hepatocellular carcinoma; ROC curve: receiver operating characteristic curve; AUC: the area under the receiver operating characteristic curve.

To validate the clinical application of the CLIA, serum CHI3L1 levels were detected in samples from 82 patients with CHB and 21 healthy controls (Figure 2 and raw data in Supplementary Table 6). Compared with that in the healthy controls (median: 32.70 ng/mL, *P*=0.001) and patients with CHB without HCC (median: 38.72 ng/mL, *P*=0.001), the CHI3L1 concentrations were significantly higher in patients with CHB and HCC (median: 122.14 ng/mL, *P*=0.001, Figure 2(a)). The CHI3L1 concentrations did not change significantly with increasing liver fibrosis stages (*P*=0.767, Figure 2(b)), nor did the FIB-4(*P*=0.068) and APRI (*P*=0.055) indices. The AUC of CHI3L1 for the diagnosis of HBV-related HCC was 0.808, with a sensitivity of 76.19% and specificity of 80.49% at a cutoff of 51.00 ng/mL (Figure 2(c)). As shown in Table 5, we found that there were significant correlations between CHI3L1 and AST (*r* = 0.284, *P*=0.005), ALP (*r* = 0.374, *P*=0.001), AFP (*r* = 0.694, *P*=0.001), LDH (*r* = 0.431, *P*=0.001), FIB-4 (*r* = 0.638, *P*=0.001), and APRI (*r* = 0.543, *P*=0.001), while no correlation was observed between CHI3L1 and the other clinical indicators.

## 4. Discussion

In the present study, we evaluated a simple and rapid CLIA for the quantitative detection of serum CHI3L1, which presented good analytical performance, making it valuable for the diagnosis of HBV-related HCC.

Currently, CLIAs are increasingly being used in the biological analysis because of their extreme sensitivity, high specificity, efficient simplified detection procedures, and short assay time [13]. Therefore, we evaluated the performance of a CLIA to detect serum CHI3L1 according to the CLSI guidelines. The assay kit showed good linearity at CHI3L1 concentrations ranging from 1.50 to 2000.00 ng/mL, with a lower LOD of 0.98 ng/mL (Tables 1 and 2). It has been reported that the upper pathophysiological concentration range of CHI3L1 can exceed 1000.00 ng/mL [21]. In this case, the CLIA for CHI3L1 detection had the advantage of a wide linear range compared with other detection methods (Table 2). Its low background signal meant that the sensitivity of CLIA for CHI3L1 detection was higher than other detection technologies (Table 2). Compared with the widely applied ELISA method, the CLIA assay greatly reduces the detection time because it provides a simplified procedure. However, the available equipment and costs of the CLIA assay should be considered in primary hospitals. In the precision assay, the intraassay, interassay, and total precision CVs were all below 10.00% (Table 4). Compared with previously reported methods [10–12], CLIA presented a simple operation process and shorter detection time, which was the main reason for its excellent precision and avoidance of unpredictable variations. In addition, it has been reported that the CHI3L1 level is approximately 40 ng/mL in healthy blood serum [22], which was consistent with our data (Figure 2(a)). Notably, the CLIA method is highly automated, making it suitable for the detection of CHI3L1 in large-scale clinical samples. The CLIA method to detect CHI3L1 represents a powerful tool to further explore the clinical value of CHI3L1.

Liver diseases are commonly caused by virus infections, especially HBV infections, in China [1]. Considering the important clinical value of CHI3L1, we evaluated the CLIA for serum CHI3L1 detection in HBV-related liver diseases. From the baseline clinical characteristics, no pregnant or lactating women were found at the time of sample exclusion. In addition, we found that HBV infection predominantly occurred in males (71/103), which might be due to their poorer lifestyle habits, such as alcohol consumption and smoking, compared with women [23]. The CHI3L1 concentrations were significantly higher in patients with CHB and HCC than those in the healthy controls and patients with CHB without HCC (Figure 2(a)), which was consistent with the results obtained by Jiang et al. [7] and Liang [24]. Studies have shown that CHI3L1 plays critical roles in cancer cell growth, proliferation, invasion, metastasis, angiogenesis, and immunoregulation [3, 25, 26]. These features might account for the high CHI3L1 levels in HCC; therefore, the effect of CHI3L1 on the occurrence and development of HCC should be further studied. We also observed a three-fold increase in the median level of CHI3L1 in patients with CHB and HCC (Figure 2(a)). This distinct differential expression suggested that CHI3L1 might be highly specific in HBV-related HCC. Indeed, compared with other clinical indicators, both AFP and CHI3L1 had a moderate diagnostic value for HBV-related HCC (Table 5). Serum AFP is a noninvasive marker commonly used in clinical laboratory screening for HCC. Meanwhile, the highest significant correlation was between CHI3L1 and AFP (Table 5, *r* = 0.694, *P* < 0.001). These results indicated that CHI3L1 has a good diagnostic value in HBV-related HCC. CHI3L1 concentrations did not change significantly with the increase in liver fibrosis stages, nor did the FIB-4 and APRI indices, and all three showed poor diagnostic value for liver fibrosis (Figure 2(b)). However, serum CHI3L1, FIB-4, and APRI were recommended indicators to assess liver fibrosis [9, 20]. The main reason for this discrepancy might be that the number of patients was small, especially the sample size of patients at the stage S3-S4 fibrosis (19/61). The early stages of liver fibrosis can be reversible with treatment. Otherwise, liver fibrosis can lead to life-threatening LC or HCC [9]. Therefore, it is necessary to further explore CHI3L1 for the diagnosis of liver fibrosis, including large-scale and multicenter studies, multifactor analysis, and the combined application of multiple indicators or methods. CHI3L1 can be used as a biomarker for prognostic assessment and targeted therapy in liver diseases [27, 28], which also needs to be validated by using the CLIA. Considering the important clinical value of CHI3L1 in other diseases [3], the developed CLIA will be a powerful tool to further explore the clinical relevance of CHI3L1.

## 5. Conclusions

In conclusion, we validated the CLIA for the rapid determination of serum CHI3L1 levels in HBV-related liver diseases. Compared with the widely applied ELISA, the CLIA assay greatly reduces the detection time. In addition, the CLIA assay has a high sensitivity, a wide linear range, and an acceptable accuracy and precision for the quantitative detection of CHI3L1, which meets the requirements for clinical testing. The CLIA assay for CHI3L1 detection also showed a good diagnostic performance in HBV-related HCC. This CLIA represents a powerful tool to further explore the clinical value of CHI3L1.

## Figures and Tables

**Table 1:** Analysis of the CHI3L1 linear range.

Dilution ratio (L : H)	Expected concentration (ng/mL)	Measured value (ng/mL)		Detection average (ng/mL)
		1st	2nd	
5 : 0	1.53	1.52	1.54	1.53
4 : 1	398.42	395.42	395.41	395.42
3 : 2	795.32	775.26	775.41	775.34
2 : 3	1192.21	1145.00	1125.00	1135.00
1 : 4	1589.11	1524.00	1527.00	1525.50
0 : 5	1986.00	1985.00	1987.00	1986.00

L, low; H, high; CHI3L1, chitinase-3-like protein 1.

**Table 2:** Immunoassay methods to detect serum CHI3L1 levels.

Methods	ELISA	MB-FIA	FICA	CLIA
Time (min)	>120	>20	>20	>20
Detection range (ng/mL)	60.35–969.98	2.90–111.00	5.00–200.00	0.98–2000.00
LOD (ng/mL)	60.35	2.90	5.00	0.98
Reference	2016 [10]	2015 [11]	2021 [12]	Present study

ELISA, enzyme-linked immunosorbent assay; MB-FIA, magnetic bead fluorescent immunoassay; FICA, fluorescence immunochromatography assay; CLIA, chemiluminescence immunoassay; LOD, limit of detection; CHI3L1, chitinase-3-like protein 1.

**Table 3:** Evaluation of accuracy.

Calibration solution	Instrumental concentration (ng/mL)	Theoretical concentration (ng/mL)	Bias (%)
Low CHI3L1 concentration	71.24	70.90	0.48
High CHI3L1 concentration	864.20	864.90	0.08

CHI3L1, chitinase-3-like protein 1. Bias (%) = ((instrumental concentration − theoretical concentration)/theoretical concentration) × 100%.

**Table 4:** Evaluation of precision.

Level	Mean (ng/mL)	Intraassay precision		Interassay precision		Total precision	
		SD	CV (%)	SD	CV (%)	SD	CV (%)
Level 1	19.73	0.36	1.81	1.53	7.76	1.56	7.91
Level 2	85.27	2.35	2.76	1.37	1.61	2.46	2.88

SD, standard deviation; CV, coefficient of variation. CV (%) = (SD/mean) × 100%.

**Table 5:** Comparison of serum CHI3L1 and clinical indicators.

Indicators	CHI3L1	ALT	AST	ALP	GGT	TP	TBIL	AFP	LDH	FIB-4	APRI
*Diagnostic value*											
AUC (HCC)	0.808	0.507	0.748	0.698	0.723	0.614	0.522	0.836	0.767	0.884	0.821
AUC (LC)	0.686	0.510	0.589	0.570	0.587	0.552	0.566	0.600	0.516	0.758	0.731

*Correlation analysis*											
r (CH I3L1)	—	0.032	0.284	0.374	0.074	0.049	0.007	0.694	0.431	0.638	0.543
*P*	—	0.752	0.005	0.001	0.470	0.630	0.949	0.001	0.001	0.001	0.001

CHI3L1, chitinase-3-like protein 1; ALT: alanine aminotransferase; AST: aspartate aminotransferase; ALP, alkaline phosphatase; GGT, glutamyl endopeptidase; TP, total protein; TBIL, total bile acid; AFP, *α*-fetoprotein; LDH, lactate dehydrogenase; FIB-4, fibrosis-4; APRI, aspartate aminotransferase-to-platelet ratio index; AUC, the area under the receiver operating characteristic curve; HCC, hepatic carcinoma; LC, liver cirrhosis.

## Data Availability

The data used to support the findings of the study are available upon request.

## Supplementary Materials

Supplementary Materials The supplementary data used to support the findings of this study are included within the supplementary information file. Supplementary Table 1: the raw data used to construct the CHI3L1 calibration curve. Supplementary Table 2: the raw data used to evaluate the limit of detection. Supplementary Table 3: the instrumental concentrations used in the evaluation of accuracy. Supplementary Table 4: the raw data used to evaluate precision. Supplementary Table 5: data used for the validation of the biological reference intervals. Supplementary Table 6: clinical data.

## References

[1] Trepo C., Chan H. L. Y., Lok A. (2014). Hepatitis B virus infection. *The Lancet*.

[2] Chinese Society Of Infectious Diseases (2019). Guidelines on the prevention and treatment in chronic hepatitis B. *Chinese Journal of Clinical Hepatology*.

[3] Zhao T., Su Z., Li Y., Zhang X., You Q. (2020). Chitinase-3 like-protein-1 function and its role in diseases. *Signal Transduction and Targeted Therapy*.

[4] Majewski S., Szewczyk K., Jerczyńska H. (2022). Longitudinal and comparative measures of serum chitotriosidase and YKL-40 in patients with idiopathic pulmonary fibrosis. *Frontiers in Immunology*.

[5] Wang S., Hu M., Qian Y. (2020). CHI3L1 in the pathophysiology and diagnosis of liver diseases. *Biomedicine & Pharmacotherapy*.

[6] Huang H., Wu T., Mao J. (2015). CHI3L1 is a liver-enriched, noninvasive biomarker that can Be used to stage and diagnose substantial hepatic fibrosis. *OMICS: A Journal of Integrative Biology*.

[7] Jiang Z., Wang S., Jin J. (2020). The clinical significance of serum chitinase 3‐like 1 in hepatitis B–related chronic liver diseases. *Journal of Clinical Laboratory Analysis*.

[8] Chinese Society Of Hepatology (2018). Guidelines on the management of hepatic encephalopathy in cirrhosis. *Zhonghua Gan Zang Bing Za Zhi*.

[9] Huang X., Zhuang J., Yang Y. (2022). Diagnostic value of serum chitinase-3-like protein 1 for liver fibrosis: a meta-analysis. *BioMed Research International*.

[10] Xie E., Zhang Q., Ma J. (2016). Evaluation of chitosinase 3-like protein 1 and its preliminary application in the diagnosis of hepatocellular carcinoma. *Journal Of Practical Medicine*.

[11] Schmalenberg M., Beaudoin C., Bulst L., Steubl D., Luppa P. B. (2015). Magnetic bead fluorescent immunoassay for the rapid detection of the novel inflammation marker YKL40 at the point-of-care. *Journal of Immunological Methods*.

[12] Pan J. (2021). Performance evaluation of fluorescence immunochromatography for quantitative detection of CHI3L1 and its application in diagnosis of liver fibrosis. *China Medical Devic Information*.

[13] Ping Y., Wang X., Dai Y. (2021). A quantitative detection of Cardiotrophin‐1 in chronic heart failure by chemiluminescence immunoassay. *Journal of Clinical Laboratory Analysis*.

[14] Clinical and Laboratory Standards Institute (2003). *EP6-A: Evaluation of the Linearity of Quantitative Measurement Procedures: A Statistical Approach*.

[15] Clinical and Laboratory Standards Institute (2000). *EP17-A: Protocols Determination of Limits of Detection and Limits of Quantitation*.

[16] Clinical and Laboratory Standards Institute (2014). *EP10-A3: Preliminary Evaluation of Quantitative Clinical Laboratory Measurement Procedures*.

[17] Clinical and Laboratory Standards Institute (2006). *EP15-A2: User Verification of Performance for Precision and Trueness; Approved Guideline*.

[18] Clinical and Laboratory Standards Institute (2010). *EP28-A3c: Defining Establishing and Verifying Reference Intervals in the Clinical Laboratory*.

[19] Clinical and Laboratory Standards Institute (2000). *EP9-A: Method Comparison and Bias Estimation Using Patient Samples; Approved Guideline*.

[20] Huang D., Lin T., Wang S. (2019). The liver fibrosis index is superior to the APRI and FIB-4 for predicting liver fibrosis in chronic hepatitis B patients in China. *Brihanmumbai Municipal Corporation Infectious Diseases*.

[21] Kronborg G., Ostergaard C., Weis N. (2002). Serum level of YKL-40 is elevated in patients with Streptococcus pneumoniae bacteremia and is associated with the outcome of the disease. *Scandinavian Journal of Infectious Diseases*.

[22] Bojesen S. E., Johansen J. S., Nordestgaard B. G. (2011). Plasma YKL-40 levels in healthy subjects from the general population. *Clinica Chimica Acta*.

[23] Qin L. (2019). Epidemiological characteristics spatial analysis of hepatitis B in Nanning.

[24] Liang S. (2019). Clinical value of serum chitinase-3-like protein 1 detection in HBV-related chronic liver diseases.

[25] Qiu Q. C., Wang L., Jin S. S. (2018). CHI3L1 promotes tumor progression by activating TGF-*β* signaling pathway in hepatocellular carcinoma. *Scientific Reports*.

[26] Yeo J., Lee C.-K., Han S.-B., Yun J., Jin T. H. (2019). Roles of chitinase 3-like 1 in the development of cancer, neurodegenerative diseases, and inflammatory diseases. *Pharmacology & Therapeutics*.

[27] Wang S., Chen S., Jin M. (2022). Diagnostic and prognostic value of serum Chitinase 3‐like protein 1 in hepatocellular carcinoma. *Journal of Clinical Laboratory Analysis*.

[28] Johansen J. S., Jensen B. V., Roslind A., Price P. A. (2007). Is YKL-40 a new therapeutic target in cancer?. *Expert Opinion on Therapeutic Targets*.

