# Supplementary material for: Analytical and Clinical Evaluation of a Chemiluminescent Immunoassay to Detect Serum Chitinase-3-like Protein 1 in HBV-Related Liver Diseases
Source: Int J Anal Chem. 2024 Jan 24;2024:6688819. doi: 10.1155/2024/6688819 (PMC10830935; doi:10.1155/2024/6688819)
Supplement: Supplementary Materials — The supplementary data used to support the findings of this study are included within the supplementary information file. Supplementary Table 1: the raw data used to construct the CHI3L1 calibration curve. Supplementary Table 2: the raw data used to evaluate the limit of detection. Supplementary Table 3: the instrumental concentrations used in the evaluation of accuracy. Supplementary Table 4: the raw data used to evaluate precision. Supplementary Table 5: data used for the validation of the biological reference intervals. Supplementary Table 6: clinical data. [file 6688819.f1.doc]

**Supplementary Table 1 The** **raw data of CHI3L1 calibration curve**

| Calibration | CHI3L1 concentration(ng/ml) | Chemiluminescence signal | | |
| --- | --- | --- | --- | --- |
|  |  | 1st | 2nd | Mean |
| S0 | 0.000 | 85 | 83 | 84 |
| S1 | 80.050 | 11585 | 11797 | 11691 |
| S2 | 1070.480 | 145173 | 148344 | 146758 |

S, calibration solutions; CHI3L1, chitinase-3-like protein 1.

**Supplementary Table 2 The raw data in** **evaluation of limit of detection**

| Calibration | Chemiluminescence signal | | | | | | | | | | | | |
| --- | --- | --- | --- | --- | --- | --- | --- | --- | --- | --- | --- | --- | --- |
|  | 1 | 2 | 3 | 4 | 5 | 6 | 7 | 8 | 9 | 10 | Mean | SD | Mean+2SD |
| S0 | 148 | 148 | 145 | 157 | 187 | 154 | 159 | 152 | 175 | 174 | 159.90 | 14.04 | 187.97 |

S, calibration solutions; SD, standard deviation; LOD, limit of detection.

**Supplementary Table 3 The instrumental concentration in** **evaluation of accuracy**

| Calibration solution | Instrumental concentration (ng/mL) | | | | | | | | | |
| --- | --- | --- | --- | --- | --- | --- | --- | --- | --- | --- |
|  | 1 | 2 | 3 | 4 | 5 | 6 | 7 | 8 | 9 | 10 |
| Low CHI3L1 concentration | 71.21 | 71.25 | 71.45 | 71.24 | 71.96 | 72.12 | 71.24 | 70.26 | 70.45 | 71.18 |
| High CHI3L1 concentration | 862.45 | 862.15 | 864.32 | 868.36 | 867.25 | 864.25 | 862.25 | 865.25 | 864.45 | 861.25 |

CHI3L1, chitinase-3-like protein 1.

**Supplementary Table 4 The raw data in evaluation of precision**

| Repeated times | Level 1 | | | | | Level 2 | | | | |
| --- | --- | --- | --- | --- | --- | --- | --- | --- | --- | --- |
|  | Day1 | Day2 | Day3 | Day4 | Day5 | Day1 | Day2 | Day3 | Day4 | Day5 |
| 1st | 17.44 | 19.69 | 19.25 | 20.00 | 21.52 | 85.76 | 89.56 | 82.11 | 87.75 | 82.11 |
| 2nd | 17.85 | 19.17 | 19.90 | 20.41 | 21.58 | 85.79 | 82.14 | 84.27 | 87.07 | 84.27 |
| 3rd | 17.06 | 19.40 | 20.00 | 20.96 | 21.74 | 85.75 | 82.14 | 86.40 | 87.56 | 86.40 |

**Supplementary Table 5 The raw data of reference intervals**

| Patient number | 1 | 2 | 3 | 4 | 5 | 6 | 7 | 8 | 9 | 10 | 11 | 12 | 13 | 14 | 15 | 16 | 17 | 18 | 19 | 20 | 21 |
| --- | --- | --- | --- | --- | --- | --- | --- | --- | --- | --- | --- | --- | --- | --- | --- | --- | --- | --- | --- | --- | --- |
| CHI3L1 | 7.53 | 26.86 | 19.25 | 27.53 | 21.58 | 33.02 | 37.86 | 26.98 | 49.43 | 44.17 | 32.70 | 40.18 | 25.43 | 28.96 | 39.43 | 36.13 | 43.15 | 25.38 | 31.87 | 33.80 | 39.20 |

**Supplementary Table 6 Clinical data**

| Number | Gender | Age | Health status | Group | CHI3L1 | ALT | AST | ALP | GGT | TP | TBIL | AFP | LDH | PLT | FIB4 | APRI |
| --- | --- | --- | --- | --- | --- | --- | --- | --- | --- | --- | --- | --- | --- | --- | --- | --- |
| 1 | male | 66 | 0 | 0 | 7.53 | 18.1 | 17.5 | 72 | 50 | 71.8 | 11.6 | 1.89 | / | 168 | 1.616 | 0.260 |
| 2 | female | 51 | 0 | 0 | 26.86 | 17.7 | 22.9 | 84 | 34 | 71.8 | 6.1 | 1.90 | 182 | 361 | 0.769 | 0.159 |
| 3 | male | 91 | 0 | 0 | 19.25 | 16.4 | 19.3 | 98 | 62 | 61.9 | 5.3 | 2.41 | 162 | 289 | 1.501 | 0.167 |
| 4 | female | 25 | 0 | 0 | 27.53 | 39.3 | 22.8 | 81 | 48 | 55.6 | 7.2 | / | 179 | 209 | 0.435 | 0.273 |
| 5 | male | 69 | 0 | 0 | 21.58 | 26.8 | 13.3 | 82 | 258 | 63.1 | 12.9 | 2.39 | 147 | 261 | 0.679 | 0.127 |
| 6 | female | 39 | 0 | 0 | 33.02 | 10.2 | 15.4 | 37 | 15 | 81.1 | 7.3 | 4.28 | / | 254 | 0.740 | 0.152 |
| 7 | female | 47 | 0 | 0 | 37.86 | 17.6 | 14.1 | 56 | 19 | 64.9 | 7.2 | / | / | 307 | 0.515 | 0.115 |
| 8 | male | 81 | 0 | 0 | 26.98 | 13.1 | 17.5 | 74 | 12 | 64.4 | 5.7 | / | 211 | 155 | 2.527 | 0.282 |
| 9 | female | 69 | 0 | 0 | 49.43 | 10.4 | 12.2 | 88 | 16 | 69.6 | 4.5 | 4.89 | 357 | 283 | 0.922 | 0.108 |
| 10 | female | 48 | 0 | 0 | 44.17 | 9.8 | 11.8 | 128 | 53 | 59.3 | 2.7 | 1.89 | 474 | 271 | 0.668 | 0.109 |
| 11 | male | 43 | 0 | 0 | 32.70 | 9.5 | 12.6 | 74 | 15 | 67.2 | 6.2 | 3.53 | 129 | 146 | 1.204 | 0.216 |
| 12 | male | 63 | 0 | 0 | 40.18 | 14.7 | 15.5 | 55 | 15 | 67.2 | 9.1 | 2.60 | 135 | 277 | 0.919 | 0.140 |
| 13 | female | 82 | 0 | 0 | 25.43 | 34.9 | 27.1 | 106 | 42 | 64.2 | 10.8 | / | 186 | 197 | 1.909 | 0.344 |
| 14 | female | 88 | 0 | 0 | 28.96 | 21.3 | 21.0 | 73 | 30 | 60.1 | 15.9 | 2.31 | 193 | 296 | 1.353 | 0.177 |
| 15 | male | 55 | 0 | 0 | 39.43 | 34.1 | 25.1 | 67 | 48 | 69.9 | 15.8 | 1.89 | 140 | 224 | 1.055 | 0.280 |
| 16 | male | 78 | 0 | 0 | 36.13 | 19.2 | 19.7 | 69 | 43 | 60.7 | 4.1 | 1.95 | 171 | 149 | 2.354 | 0.331 |
| 17 | female | 75 | 0 | 0 | 43.15 | 21.3 | 24.1 | 68 | 8 | 59.4 | 7.9 | 2.17 | / | 208 | 1.883 | 0.290 |
| 18 | male | 53 | 0 | 0 | 25.38 | 14.9 | 14.1 | 62 | 20 | 63.5 | 3.3 | / | 135 | 321 | 0.603 | 0.110 |
| 19 | female | 94 | 0 | 0 | 31.87 | 14.3 | 28.2 | 97 | 8 | 61.7 | 10.4 | / | 262 | 141 | 4.972 | 0.500 |
| 20 | female | 87 | 0 | 0 | 33.80 | 8.1 | 14.6 | 52 | 8 | 48.2 | 6.9 | 2.86 | 368 | 194 | 2.301 | 0.188 |
| 21 | female | 57 | 0 | 0 | 39.20 | 20.3 | 18.5 | 53 | 11 | 66.6 | 8.3 | 2.81 | 137 | 187 | 1.252 | 0.247 |
| 22 | female | 36 | 4 | 1 | 7.53 | 17.0 | 14.7 | 43 | 20 | 63.7 | 5.0 | 1.89 | 154 | 279 | 0.460 | 0.132 |
| 23 | male | 48 | 4 | 1 | 10.20 | 23.3 | 16.1 | 52 | 24 | 77.0 | / | 2.88 | 141 | 177 | 0.905 | 0.227 |
| 24 | male | 39 | 1 | 1 | 12.65 | 34.4 | 32.7 | 134 | 9 | 50.8 | 148.4 | 1.89 | / | 210 | 1.035 | 0.389 |
| 25 | male | 25 | 4 | 1 | 13.44 | 18.5 | 22.9 | 84 | 9 | 75.6 | 10.8 | 2.27 | 157 | 177 | 0.752 | 0.323 |
| 26 | male | 39 | 2 | 1 | 13.85 | 16.2 | 14.1 | 67 | 19 | 68.1 | 18.9 | 1.89 | 151 | 215 | 0.635 | 0.164 |
| 27 | male | 35 | 3 | 1 | 14.06 | 21.5 | 22.2 | 62 | 18 | 77.2 | 15.0 | 2.50 | 146 | 206 | 0.813 | 0.269 |
| 28 | male | 50 | 1 | 1 | 14.57 | 23.0 | 19.7 | 51 | 15 | 65.4 | 10.0 | 2.17 | 112 | 214 | 0.960 | 0.230 |
| 29 | male | 57 | 4 | 1 | 15.17 | 26.0 | 15.3 | 53 | 19 | 69.1 | 18.5 | 3.72 | 132 | 132 | 1.296 | 0.290 |
| 30 | male | 35 | 1 | 1 | 15.40 | 18.0 | 14.9 | 46 | 15 | 66.5 | 7.9 | 3.23 | 126 | 285 | 0.431 | 0.131 |
| 31 | male | 34 | 2 | 1 | 15.69 | 21.6 | 21.2 | 63 | 18 | 72.5 | 22.0 | 2.23 | 154 | 184 | 0.843 | 0.288 |
| 32 | male | 34 | 1 | 1 | 16.40 | 26.0 | 18.2 | 79 | 35 | 79.0 | 26.0 | 3.13 | 140 | 289 | 0.420 | 0.157 |
| 33 | male | 47 | 3 | 1 | 17.75 | 13.2 | 13.6 | 65 | 24 | 71.0 | 6.1 | 2.32 | 146 | 281 | 0.626 | 0.121 |
| 34 | male | 59 | 5 | 2 | 19.25 | 74.1 | 101.2 | 263 | 401 | 72.2 | 12.7 | 16.49 | 203 | 233 | 2.977 | 1.086 |
| 35 | female | 57 | 1 | 1 | 19.90 | 15.8 | 21.5 | 74 | 12 | 73.5 | 8.2 | 3.27 | 155 | 222 | 1.389 | 0.242 |
| 36 | male | 41 | 1 | 1 | 20.00 | 20.2 | 14.0 | 87 | 16 | 74.9 | 9.2 | 3.09 | / | 267 | 0.478 | 0.131 |
| 37 | female | 49 | 1 | 1 | 20.00 | / | / | / | / | / | / | 1.89 | / | 150 | / | 0.000 |
| 38 | male | 46 | 2 | 1 | 20.41 | 22.1 | 34.1 | 54 | 11 | 67.0 | 13.1 | 5.39 | 158 | 150 | 2.224 | 0.568 |
| 39 | female | 46 | 1 | 1 | 20.96 | 45.7 | 32.5 | 42 | 19 | 63.7 | 14.2 | 3.59 | 147 | 205 | 1.079 | 0.396 |
| 40 | male | 20 | 1 | 1 | 21.52 | 27.7 | 23.3 | 79 | 19 | 73.2 | 8.2 | 1.89 | 167 | 201 | 0.441 | 0.290 |
| 41 | male | 29 | 4 | 1 | 21.74 | 23.1 | 18.2 | 41 | 12 | 75.2 | 15.4 | 1.89 | 143 | 238 | 0.461 | 0.191 |
| 42 | female | 46 | 1 | 1 | 21.99 | 23.1 | 15.4 | 37 | 12 | 71.9 | / | / | 163 | 274 | 0.538 | 0.141 |
| 43 | male | 90 | 5 | 2 | 22.95 | 61.7 | 37.6 | 71 | 99 | 56.9 | 12.6 | 1.89 | / | 142 | 3.034 | 0.662 |
| 44 | male | 46 | 4 | 1 | 23.36 | 19.9 | 17.8 | 88 | 26 | 62.3 | 9.8 | 2.22 | 138 | 180 | 1.020 | 0.247 |
| 45 | male | 38 | 1 | 1 | 25.92 | 162.5 | 71.5 | 78 | 61 | 68.4 | 11.2 | 2.97 | 196 | 246 | 0.866 | 0.727 |
| 46 | male | 44 | 2 | 1 | 26.11 | 22.9 | 18.9 | 59 | 16 | 69.2 | 8.3 | 2.61 | 158 | 185 | 0.939 | 0.255 |
| 47 | female | 54 | 5 | 2 | 26.30 | 11.1 | 16.8 | 79 | 25 | 62.3 | 10.0 | 11.65 | 126 | 76 | 3.583 | 0.553 |
| 48 | female | 30 | 2 | 1 | 27.21 | 36.9 | 24.4 | 79 | 23 | 77.6 | 10.0 | 1.89 | 136 | 354 | 0.340 | 0.172 |
| 49 | male | 35 | 1 | 1 | 27.34 | 18.0 | 14.9 | 46 | 15 | 66.5 | 7.9 | 3.23 | 126 | 285 | 0.431 | 0.131 |
| 50 | female | 51 | 5 | 2 | 27.49 | 10.8 | 37.7 | 77 | 27 | 79.6 | 11.8 | 4.60 | 159 | 213 | 2.747 | 0.442 |
| 51 | female | 46 | 1 | 1 | 27.53 | / | / | / | / | / | / | 1.89 | 145 | 101 | / | 0.000 |
| 52 | male | 35 | 2 | 1 | 31.68 | 73.4 | 34.5 | 54 | 43 | 73.3 | 13.9 | 3.98 | 168 | 219 | 0.644 | 0.394 |
| 53 | female | 36 | 2 | 1 | 32.70 | 11.9 | 17.7 | 53 | 10 | 78.9 | / | 3.66 | / | 394 | 0.469 | 0.112 |
| 54 | male | 43 | 5 | 2 | 33.02 | 34.5 | 33.1 | 83 | 46 | 71.7 | 6.2 | 21.16 | 199 | 275 | 0.881 | 0.301 |
| 55 | male | 43 | 4 | 1 | 34.37 | 19.7 | 20.4 | 78 | 34 | 69.2 | 12.3 | 1.89 | 138 | 233 | 0.848 | 0.219 |
| 56 | female | 52 | 2 | 1 | 36.13 | 23.8 | 27.9 | 77 | 89 | 76.6 | 9.0 | 2.35 | / | 189 | 1.573 | 0.369 |
| 57 | male | 44 | 4 | 1 | 38.72 | 15.8 | 19.6 | 62 | 21 | 62.6 | 14.3 | 1.89 | 117 | 87 | 2.494 | 0.563 |
| 58 | male | 42 | 2 | 1 | 38.88 | 18.5 | 18.6 | 45 | 9 | 68.1 | 10.5 | 1.89 | 166 | 139 | 1.307 | 0.335 |
| 59 | male | 26 | 4 | 1 | 39.20 | 29.4 | 16.8 | 51 | 27 | 68.0 | 11.7 | 1.89 | 165 | 172 | 0.468 | 0.244 |
| 60 | male | 51 | 1 | 1 | 40.27 | / | / | / | / | / | / | 2.76 | / | 216 | / | 0.000 |
| 61 | female | 46 | 1 | 1 | 42.79 | 23.1 | 15.4 | 37 | 12 | 71.9 | / | / | 163 | 274 | 0.538 | 0.141 |
| 62 | male | 42 | 3 | 1 | 43.15 | 145.2 | 100.4 | 108 | 73 | 82.3 | 15.9 | 46.67 | / | 164 | 2.134 | 1.530 |
| 63 | male | 66 | 4 | 1 | 44.17 | 20.2 | 21.4 | 46 | 18 | 63.7 | 10.2 | 2.21 | 124 | 129 | 2.436 | 0.415 |
| 64 | male | 31 | 1 | 1 | 45.42 | 22.6 | 17.4 | 48 | 20 | 66.4 | 10.2 | 1.89 | 130 | 207 | 0.548 | 0.210 |
| 65 | male | 38 | 2 | 1 | 45.96 | 70.5 | 31.6 | 63 | 35 | 70.7 | 11.5 | 2.93 | 134 | 219 | 0.653 | 0.361 |
| 66 | male | 44 | 4 | 1 | 47.68 | 38.8 | 26.6 | 111 | 16 | 73.1 | 16.6 | 1.89 | 143 | 161 | 1.167 | 0.413 |
| 67 | male | 30 | 2 | 1 | 48.50 | 635.8 | 291.2 | 94 | 83 | 68.9 | 27.0 | 10.93 | 232 | 185 | 1.873 | 3.935 |
| 68 | female | 62 | 2 | 1 | 49.14 | 18.0 | 25.0 | 59 | 11 | 69.9 | 2.7 | 5.87 | 353 | 226 | 1.617 | 0.277 |
| 69 | male | 35 | 1 | 1 | 49.43 | 189.4 | 82.2 | 52 | 27 | 69.1 | 6.3 | 3.35 | 161 | 264 | 0.792 | 0.778 |
| 70 | male | 31 | 2 | 1 | 49.57 | 94.4 | 63.1 | 65 | 52 | 71.0 | 12.6 | 10.69 | 191 | 234 | 0.860 | 0.674 |
| 71 | male | 46 | 1 | 1 | 50.38 | 45.7 | 32.5 | 42 | 19 | 63.7 | 14.2 | 3.59 | 147 | 202 | 1.095 | 0.402 |
| 72 | male | 57 | 5 | 2 | 51.61 | 14.6 | 25.4 | 65 | 24 | 73.0 | 10.5 | 4.86 | 202 | 109 | 3.476 | 0.583 |
| 73 | male | 36 | 2 | 1 | 53.01 | 104.6 | 44.4 | 71 | 46 | 72.7 | 13.1 | 1.89 | 246 | 178 | 0.878 | 0.624 |
| 74 | male | 55 | 5 | 2 | 54.25 | 21.7 | 19.6 | 58 | 11 | 62.8 | 8.5 | 2.10 | 178 | 163 | 1.420 | 0.301 |
| 75 | male | 35 | 4 | 1 | 55.29 | 44.2 | 26.5 | 46 | 28 | 69.6 | 13.8 | 3.63 | 165 | 100 | 1.395 | 0.663 |
| 76 | male | 24 | 1 | 1 | 56.55 | 16.0 | 17.0 | 70 | 17 | 79.6 | 16.3 | 1.89 | 158 | 251 | 0.406 | 0.169 |
| 77 | male | 51 | 2 | 1 | 60.52 | 249.3 | 161.5 | 74 | 510 | 72.9 | 54.0 | 2.85 | 244 | 175 | 2.981 | 2.307 |
| 78 | male | 42 | 3 | 1 | 62.73 | 38.6 | 70.6 | 91 | 92 | 76.8 | 11.4 | 6.49 | / | / | / | / |
| 79 | male | 36 | 1 | 1 | 69.51 | 23.8 | 18.3 | 58 | 11 | 65.3 | 17.3 | 1.89 | 154 | 158 | 0.855 | 0.290 |
| 80 | male | 52 | 5 | 2 | 73.87 | 28.0 | 42.0 | 95 | 70 | 81.9 | 17.0 | 4.02 | 231 | 256 | 1.612 | 0.410 |
| 81 | male | 31 | 2 | 1 | 74.86 | 124.2 | 57.5 | 70 | 50 | 75.2 | 10.2 | 6.89 | 182 | 285 | 0.561 | 0.504 |
| 82 | male | 28 | 1 | 1 | 82.11 | 41.0 | 28.0 | 77 | 37 | 70.1 | 9.7 | 1.89 | / | 258 | 0.475 | 0.271 |
| 83 | male | 33 | 1 | 1 | 84.27 | 26.3 | 26.2 | 87 | 76 | 81.9 | 22.2 | 4.59 | 184 | 264 | 0.639 | 0.248 |
| 84 | male | 46 | 2 | 1 | 86.40 | 23.3 | 21.7 | 68 | 23 | 73.0 | 15.5 | 1.89 | 146 | 174 | 1.188 | 0.312 |
| 85 | female | 45 | 1 | 1 | 89.10 | 18.2 | 20.9 | 42 | 17 | 80.3 | 14.0 | 2.77 | 192 | 167 | 1.320 | 0.313 |
| 86 | male | 41 | 5 | 2 | 110.79 | 65.0 | 40.0 | 61 | 50 | 62.7 | 12.4 | 4.64 | 617 | 196 | 1.038 | 0.510 |
| 87 | male | 45 | 2 | 1 | 114.45 | 73.8 | 38.9 | 79 | 36 | 71.2 | 11.0 | 7.19 | 152 | 300 | 0.679 | 0.324 |
| 88 | male | 52 | 5 | 2 | 115.60 | 25.3 | 28.7 | 58 | 46 | 59.2 | 4.7 | 1.97 | 174 | 106 | 2.799 | 0.677 |
| 89 | male | 37 | 4 | 1 | 121.56 | 28.6 | 33.5 | 68 | 48 | 79.3 | 13.5 | 5.64 | 124 | 65 | 3.566 | 1.288 |
| 90 | male | 35 | 1 | 1 | 122.14 | 49.3 | 27.1 | 70 | 39 | 78.9 | 7.4 | 4.76 | 154 | 226 | 0.598 | 0.300 |
| 91 | male | 44 | 5 | 2 | 122.14 | 22.2 | 20.7 | 62 | 23 | 74.6 | 12.5 | 32.36 | 159 | 146 | 1.324 | 0.354 |
| 92 | female | 69 | 4 | 1 | 132.95 | 23.6 | 22.7 | 110 | 17 | 71.1 | / | 2.17 | / | / | / | / |
| 93 | male | 47 | 5 | 2 | 135.66 | 30.1 | 73.6 | 130 | 76 | 74.6 | 35.8 | 30.03 | 237 | 61 | 10.336 | 3.016 |
| 94 | male | 67 | 5 | 2 | 157.75 | 25.3 | 28.0 | 78 | 73 | 80.1 | 10.3 | 4.08 | 219 | 137 | 2.722 | 0.511 |
| 95 | male | / | 5 | 2 | 166.07 | / | / | / | / | / | / | 4.32 | / | / | / | / |
| 96 | male | / | 5 | 2 | 172.36 | / | / | / | / | / | / | 21.89 | / | / | / | / |
| 97 | female | 56 | 4 | 1 | 187.33 | 12.5 | 53.0 | 76 | 8 | 62.7 | 17.0 | 2.14 | / | 35 | 23.985 | 3.786 |
| 98 | female | 51 | 5 | 2 | 200.66 | 10.8 | 37.7 | 77 | 27 | 79.6 | 11.8 | 4.60 | 159 | 213 | 2.747 | 0.442 |
| 99 | male | 61 | 5 | 2 | 247.68 | 24.0 | / | 64 | 19 | 56.0 | 4.1 | 2.91 | 240 | 191 | / | / |
| 100 | male | 75 | 5 | 2 | 274.24 | 158.6 | / | 109 | 96 | 56.2 | / | 9.26 | 275 | 188 | / | / |
| 101 | female | 72 | 5 | 2 | 480.22 | 7.4 | 15.3 | 91 | 63 | 68.2 | 7.4 | 1120.00 | / | 79 | 5.126 | 0.484 |
| 102 | female | 65 | 5 | 2 | 485.25 | 74.2 | 122.9 | 131 | 71 | 72.6 | 26.0 | 976.40 | 460 | 60 | 15.457 | 5.121 |
| 103 | female | 79 | 5 | 2 | 556.00 | 28.1 | 112.0 | 196 | 68 | 72.9 | 15.1 | 150.80 | / | 134 | 12.456 | 2.090 |

Health status: 0, health controls; 1-4, different stages of liver fibrosis; 5, Chronic Hepatitis B with hepatic carcinoma. Group: 0, health controls; 1, Chronic Hepatitis B without hepatic carcinoma; 2,Chronic Hepatitis B with hepatic carcinoma. CHI3L1, chitinase-3-like protein 1; ALT: alanine aminotransferase; AST: aspartate aminotransferase; ALP, alkaline phosphatase; GGT, glutamyl endopeptidase; TP, total protein; TBIL, total bile acid; AFP, α-fetoprotein; LDH, lactate dehydrogenase; PLT, platelet; FIB-4, fibrosis-4; APRI, aspartate aminotransferase-to-platelet ratio index.
